# A chalcohalide glass/alloy based Ag^+^ ion - selective electrode with nanomolar detection limit

**DOI:** 10.1038/s41598-017-17032-7

**Published:** 2017-12-01

**Authors:** Lei Li, Haiwei Yin, Yang Wang, Jianhua Zheng, Huidan Zeng, Guorong Chen

**Affiliations:** School of Materials Science and Engineering, East China University of Science and Technology, Shanghai, 200237 China

## Abstract

In this paper, a silver ion-selective electrode material with lower detection limit is presented. The electrode is based on 22.5As_2_S_3_-22.5Ag_2_S-55AgCl chalcohalide glass membranes. The low detection limit decreases from the micromolar range of the original Ag_2_S-As_2_S_3_ electrode to the nanomolar level (1.89 nM) by introducing AgCl. The addition of AgCl increases the conductivity of the glasses and improves the analytical properties of electrodes because of the joint effects of Ag^+^ and Cl^-^ on network structure of the glass. The super-Nernstian response behavior was observed for the partially crystallized electrode. The electrode containing AgCl also possesses a high selectivity (except for Hg^2+^), fast response and good stability.

## Introduction

During the last four decades, much attention has been attracted to the problem of heavy metal pollution on environments and the humankind^1,2^. Heavy metals can be emitted by both natural and anthropogenic sources, including a variety of industrial resources, such as mining sites, foundries and smelters, combustion by-products and traffics^3–5^. In particular, heavy metal ions are apt to combine with the body’s biomolecules to form strong and stable chemical bonds, thus interfering with the normal metabolic processes of human beings^1^. To ensure human safety, implementation of targeted investigations on monitoring heavy metal contents in environmental solutions becomes one of the most pressing demands of the day.

To determine the heavy-metal ions (for examples Cu^2+^, Fe^3+^, Cd^2+^, Pb^2+^ and Ag^+^ etc.) in solutions, different analytical devices have been studied and developed in the past years^6–9^. Ion-selective electrodes (ISEs) are among the most effective devices due to merits of low cost, high reproducibility^10–13^ and low detection limits^14–16^. In fact, many instruments could not detect monovalent silver ions (Ag^+^) which makes Ag^+^-selective electrode become more significant. Chalcogenide (ChG) glasses represent one class of functional membranes for analysis and control of waste water or monitoring programs of rivers and lakes polluted with industrial elements and chemical industries^17,18^. Compared with their crystalline counterparts, ChG glass membranes have advantages of high selectivity, excellent lifetime and good chemical durability in strong acids and oxidizing agent media^19,20^. However, the low detection limit of the conventional Ag^+^-ISEs based on ChG glasses was only in the micromolar range^21,22^, and this has greatly restricted the application of Ag^+^-ISEs for determination of Ag^+^ ions at trace levels. It is known that halogen atoms in ChG glasses often act as a glass network terminal, and interrupt the glass network, resulting in the more open structure. Therefore, the largely improved transport property of monovalent ions is expected with addition of halides into ChG glasses^23,24^. In this paper, we try to explore the effect of AgCl addition on conductivity and ion transport performance of Ag_2_S-As_2_S_3_ glass, thereby obtaining an electrode that can be used for Ag^+^ ion trace detection.

## Materials and Methods

### Chalcohalide ChH glasses synthesis

Glasses with compositions (mol%) of (100-x)/2As_2_S_3_-(100-x)/2Ag_2_S-xAgCl were prepared by conventional melt-quenching method. The as-prepared samples are noted as G0, G45, G50, G55, and G60 for x = 0, 45, 50, 55, 60, respectively^25^. High-purity (AR) elements (As, S) and compounds (AgCl, Ag_2_S) were used as starting materials. The weighed batches (2 g) were evacuated in cleaned quartz ampoules under a vacuum of 0.10–0.01 Pa. The sealed ampoules were heated at 920 °C for 15 hrs in a rocking furnace, and then cooled down to 700 °C. The melts were finally quenched in the water from 700 °C to room temperature. The obtained glass samples were then annealed at 100 °C for 4 hrs to release the stress induced during quenching.

### Electrode preparation

The bulk glasses were cut into discs (1 mm thick and 5 mm in diameter) as electrode membranes to prepare the all-solid-state electrode. All membranes were thoroughly polished with fine abrasive powder to obtain plane-parallel plates. A silver wire was then soldered to the membranes inner surface by using conductive silver paste, while insulation coating ink was smeared on the non-working surface except one end of the silver wire and the outer surface of membranes. After that the Ag^+^-ISE was put into the drying closet until the ink being dry. The working area was about 19.6 mm^2^.

### Characterization of materials

X-ray diffraction (XRD) patterns, Raman spectra and scanning electron microscopy (SEM) were used for analysis of glass formation and structure, and electrochemical impedance spectroscopy (EIS) was measured to characterize electrical conductivity of materials. XRD patterns (MAX R1, precision ± 0.02°; Rigaku, Tokyo, Japan) were recorded on powder samples at 40 kV and 100 mA using Cu K_α_ radiation to identify the type of crystallites. Raman spectra (InVia-Reflex, precision≈1 cm^−1^, Renishaw, UK) were performed with a laser of 785 nm as the excitation source. SEM micrographs were obtained using a Hitachi S-4800 scanning electron microscope with the accelerating voltage of 1 kV. The surface of glasses was sputter coated with gold layer before measurement. EIS spectra were measured at the open circuit potential, and data were collected at a frequency range of 1MHz-1mHz.

### Electromotive force (EMF) measurements

The potentiometric measurements were performed by CHI660e Electrochemical Work Station with a two-electrode system in magnetically stirred solutions at ambient temperature (25 °C). The Ag/AgCl reference electrode was applied with 3 M KCl as reference electrolyte solution and the all-solid-state Ag^+^- ISE as a working electrode. The salt bridge with 0.1 M KNO_3_ as electrolyte was used to avoid contamination. All EMF values were corrected for liquid junction potentials according to the Henderson equation^26^ where activity coefficients were obtained from the Debye-Huckel approximation. The experiments were performed in two 50 mL beakers pretreated in 0.1 M HNO_3_. The KNO_3_ (0.1 M) was used as an ionic strength adjustor, and the constant pH value (pH = 6) of the test solution was maintained. EIS of ion-selective electrodes were measured using a three-electrode system, and a platinum electrode was used as the counter electrode.

## Results and Discussion

### Characterization of the materials

**Figure 1:**
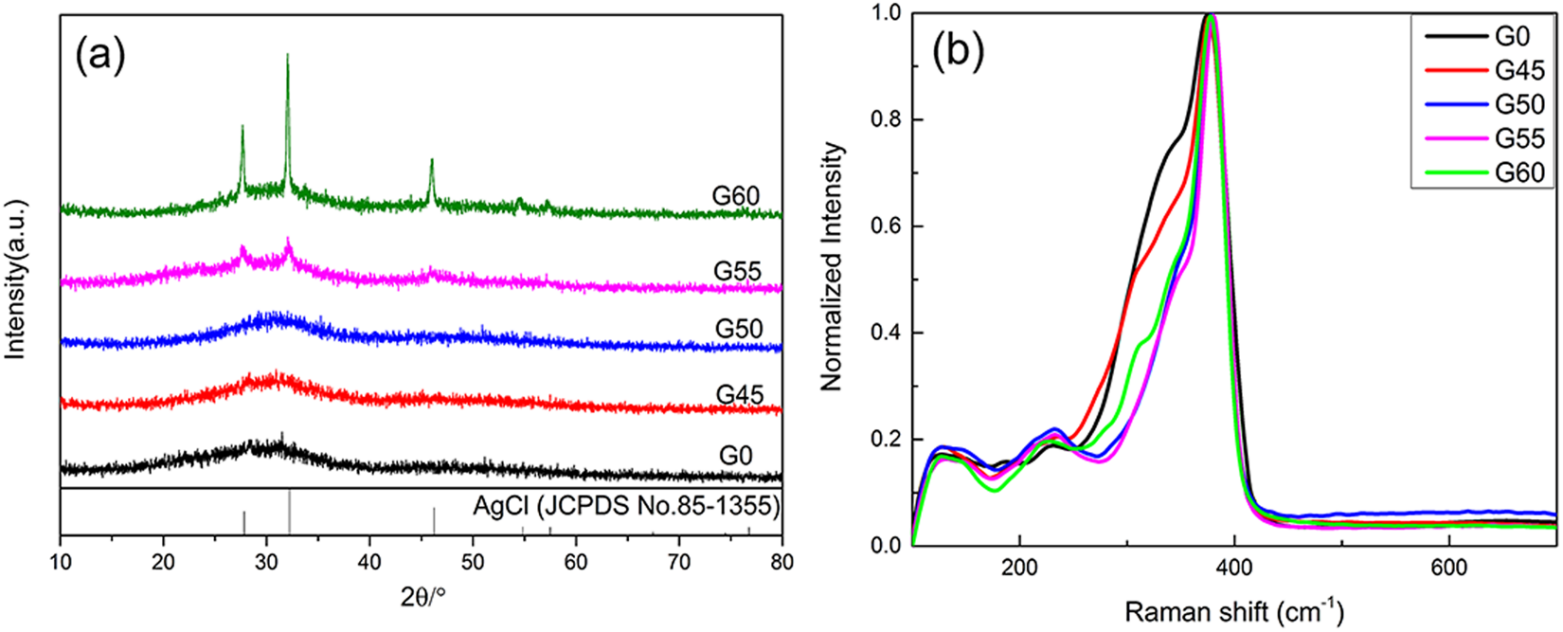
XRD patterns (**a**) and normalized Raman spectra (**b**) of all samples.

It has been reported that the optimum ratio of Ag_2_S to As_2_S_3_ in the binary As_2_S_3_-Ag_2_S glass system varied from 1:1 to 2:1 when it was used as electrode materials^27^. In the present work, the 1:1 ratio was chosen and AgCl incorporated as a network modifier. Figure 1 presents XRD patterns and Raman spectra of all as-prepared samples where ever characterized G45, G50 and G55^28^ are also included for comparison as a whole. Results demonstrate again the greater crystallization tendency of G55 relative to other samples, but its crystal diffraction peaks seem weaker compared with ref.^28^. This is because an amount of batches for the re-prepared samples in this work (2 g) was less than that in previous work (4 g)^28^ due to the reduced diameter of quartz ampoules (5 mm). Thus it allows the glass melts to have a greater contact area so as to quench in amorphous state more easily. This inevitably leads to a decrease in the glass crystallinity of G55 and accordingly the change of the Raman spectrum as to be discussed afterwards.

It is seen from Fig. 1(a) that there are broad diffraction bands for G0, G45 and G50, indicating glassy state of these samples. However, several weak crystalline peaks appear for G55 and become more intense for G60. According to the location of these XRD peaks by comparison with the JCPDS standard card as shown at the bottom of the figure, they belong to AgCl crystal phase.

Raman spectra of all samples are normalized at 375 cm^−1^ and results are shown in Fig. 1(b). For each sample, Raman spectrum was measured three times and showed similar profiles, indicating the trustworthy homogeneity of as-prepared samples. Similar to the previous reports^29,30^, the structural network of the stoichiometric glass AgAsS_2_ (G0) consists of 3-membered rings As_3_S_6_ molecular fragments, where each ring is connected to three silver atoms^29^. The band at ~375 cm^−1^ is attributed to the asymmetric stretching vibration of As-S bonds with non-bridging S atoms^30^, while two bands at ~310 cm^−1^ and ~340 cm^−1^ are assigned respectively to As-S-As vibrations of pyramidal units denoting chair like configuration of rings^31^, and the stretching vibration of As-S bonds with bridging S atoms in the AsS_3_ pyramids^32^. It is noted that the intensity of bands at 310 cm^−1^ and 340 cm^−1^ decreases gradually with the AgCl content from 0 to 55 mol% most probably due to the joint effects of Cl^−^ and Ag^+^ on the network structure of the glass. On one hand, Cl^−^ ions can act as a glass network terminal to replace some bridging S to bond with As, leading to formation of the mixed Ag_y_As[S_3-y_Cl_y_] pyramids^33^. On the other, an increase in Ag content induces more Ag-S bonds which have the larger bond length (2.6 Å) than the substituted As-S bonds (2.3 Å), making glass network more open^34^. Nevertheless, the band intensity of G60 at 310 cm^−1^ and 340 cm^−1^ increases slightly compared with G50 and G55, and this phenomenon is most likely associated with precipitation of AgCl crystals from the glass matrix, thus weakening the effect of Cl^−^ and Ag^+^ on the glass network.

**Figure 2:**
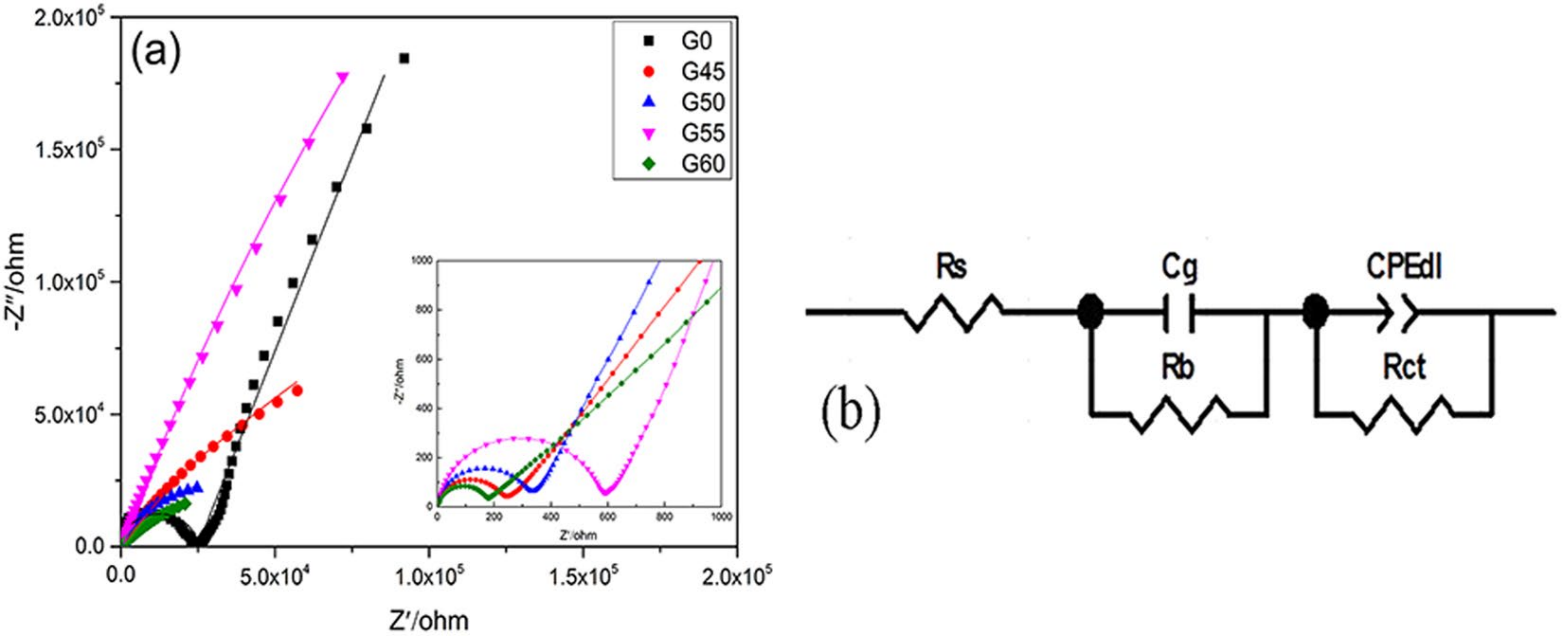
(**a**) Impedance spectra of all ISEs in 0.1 M KNO_3_ (the solid lines represent the fitting curves). Inset: partial enlargement of high frequency part. (**b**) Equivalent circuits for ISEs.

Figure 2(a) shows EIS spectra of all samples exposed to 0.1 M KNO_3_ electrolytes solution. The EIS is fitted according to the equivalent circuit^35,36^ as shown in Fig. 2(b), where R_s_ stands for solution resistance, R_b_ for membrane bulk resistance related to high frequency semicircle, C_g_ for geometric capacity of membrane, R_ct_ for charge-transfer resistance, and CPE_dl_ for constant phase element. It is clear that experimental curves (dotted lines) are consistent closely to equivalent circuit fitting curves (solid lines), while each fitting parameter has a lower fitting error (<10%), indicating the reliable fit.

Table 1 presents the fitted circuit parameters of all samples. Compared with the AgCl-free electrode (G0), the electrodes containing AgCl show the reduced bulk resistance (R_b_) by about two orders of magnitude, consistent with the expected results. It has been reported extensively that the number of carriers increases with the addition of halides in ChG and oxide systems^25,37^. Since the silver cations responsible for the transport of the current come from the ionic dissociation of Ag_2_S and AgCl in this matrix^25^, the charge carriers increase with the increased AgCl content. As demonstrated by Raman spectra in Fig. 1(b), the addition of AgCl makes the glass network more open, which is beneficial for ion migrate^24^, thus increasing the electrode conductivity. It is worth mentioning that for AgCl containing samples, the R_b_ value does increase with the increased AgCl content. Our previous studies on crystallization kinetics of G45 and G50 show that precipitated crystals experienced an evolution from AgCl (G45) to Ag_3_AsS_3_ (G50) phases after thermal treatment at the same condition^28^. It implies that the molecular fragments Ag_3_AsS_3_ in the glass matrix become dominant with the increase of AgCl content. As a result, the amount of removable Ag^+^ decreases and conductivity is accordingly reduced. In addition, crystallization is known to increase the conductivity of the glass^38^, so that G60 has the smallest R_b_ value. Similar phenomenon also occurs in the low-frequency part of the EIS spectra for samples with different AgCl content, which will be discussed later in conjunction with electrode performance.

**Table 1:** Equivalent circuit parameters of all samples.

Electrode	R_s_	C_g_	R_b_	CPE_dl_	n	R_ct_
	(Ω/cm^2^)	(nF/cm^2^)	(Ω/cm^2^)	(μF/cm^2^)		(MΩ/cm^2^)
G0	—	0.1698	24890	18.88	0.8106	3.073
G45	3.939	10.48	233.8	33.52	0.6409	0.455
G50	5.291	7.246	321.2	11.46	0.7562	0.07502
G55	6.067	3.734	587.3	16.04	0.8103	2.989
G60	3.003	13.07	161.1	110	0.538	0.137

### Dynamic response of ISEs

**Figure 3:**
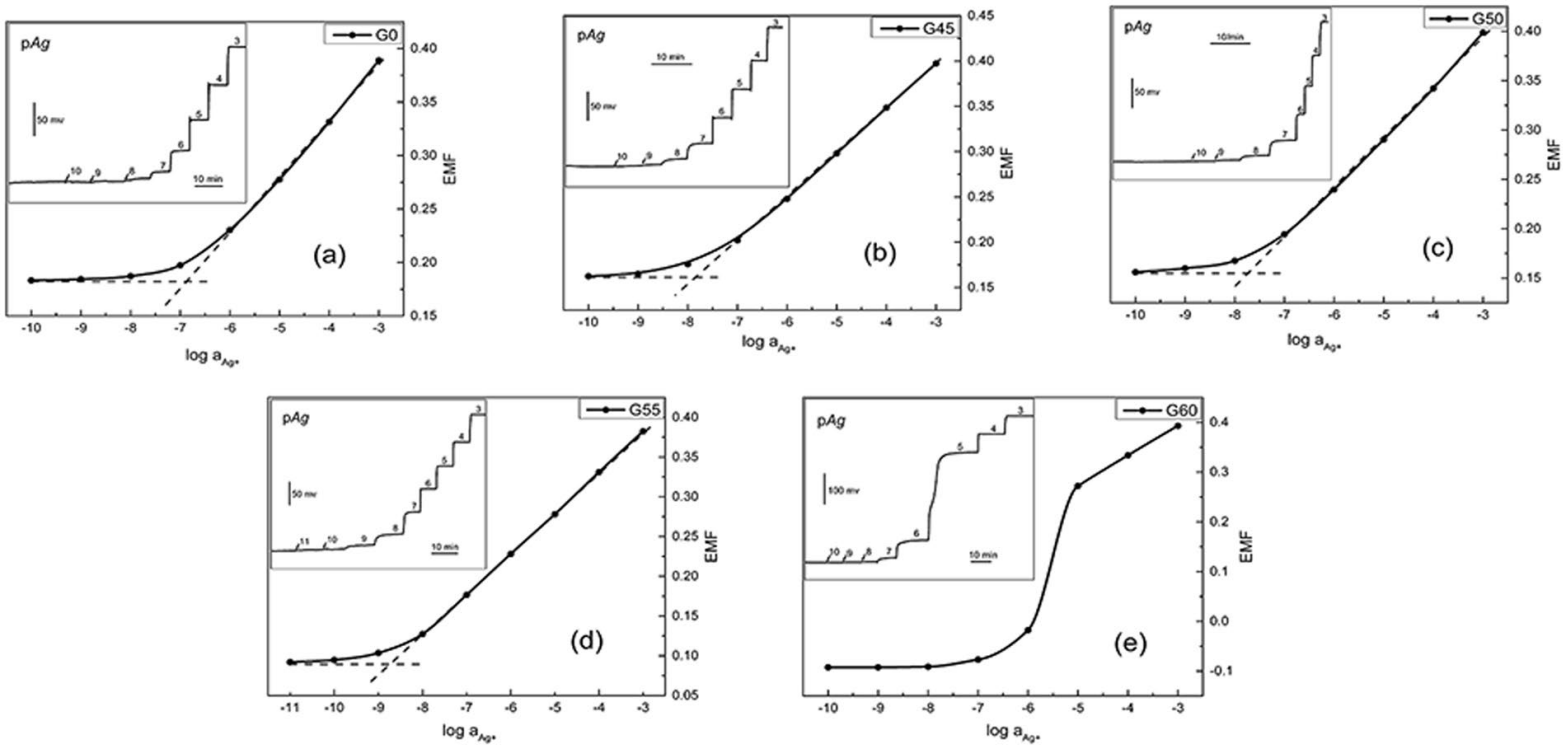
Calibration curves of all ISEs. The dotted lines are least-squares fits. Inset: time traces of the response of all ISEs.

The electrode response curve was constructed by plotting the potential values versus log a_(Ag+)_, and the calibration curves of all electrodes are shown in Fig. 3. Different concentrations of AgNO_3_ standard solutions were prepared by using deionized aqueous solution. The Ag^+^ ion concentrations for the dynamic curve test were adjusted by using a 10 μL micro-injector to add different concentrations of AgNO_3_ standard solution to a 0.1 M KNO_3_ electrolyte solution with a constant volume. According to the IUPAC recommendations, the lower detection limit can be obtained from an intersection of two straight lines of the calibration curve^39^, as shown by the dotted lines in Fig. 3. The sensitivity, linear response range and low detection limit for all samples are summarized in Table 2.

**Table 2:** Electrochemical characteristics of different ISEs.

Electrode Code	Sensitivity	Linear range	Detection limit
	(mV/pAg) (±0.5)	(mol/L) (10^±0.2^)	(nM)
G0	54.39	10^−6^–10^−3^	139(±3)
G45	52.09	10^−7^–10^−3^	14.6(±0.3)
G50	53.67	10^−7^–10^−3^	18.3(±0.3)
G55	54.47	10^−8^–10^−3^	1.89(±0.03)
G60	56.02	10^−5^–10^−3^	—

It can be seen from Table 2 that the electrochemical properties of G0 are similar to previously reported results^40^, while AgCl addition makes the analytical characteristics of G45, G50 and G55 much superior, where G55 exhibits the lowest detection limit (1.89 nM). To the best of our knowledge, this is the lowest detection limit for Ag^+^ in the ChG/ChH glass ISEs presented to date^23,40,41^. By combining with above EIS and Raman spectra, we attribute an improvement of electrode performance induced by AgCl addition to the increased conductivity and an enhanced ion migration due to a more open structure. On the other hand, G55 does not show the lowest bulk resistance (R_b_) compared with G45 and G50, indicating that conductivity is not the only factor that influences the performance of the electrode. It is known that the competition of interfering ions with the primary ions is one of reasons for the apparent loss of EMF at low Ag^+^ activity to hinder the further improvement of the low detection limit^42^. A charge-transfer resistance (R_ct_) obtained from analysis on the low-frequency part of the impedance spectrum provides information about the easiness for electron/ion transfer at the electrode-solution interface^36^. As shown in Table 1, G55 has a larger R_ct_ than G45 and G50, suggesting that the transfer of K^+^ ion at the electrode interface is difficult. Consequently, the detection limit of G55 is lower than G45 and G50 owing to the weaker K^+^ ion interference.

**Figure 4:**
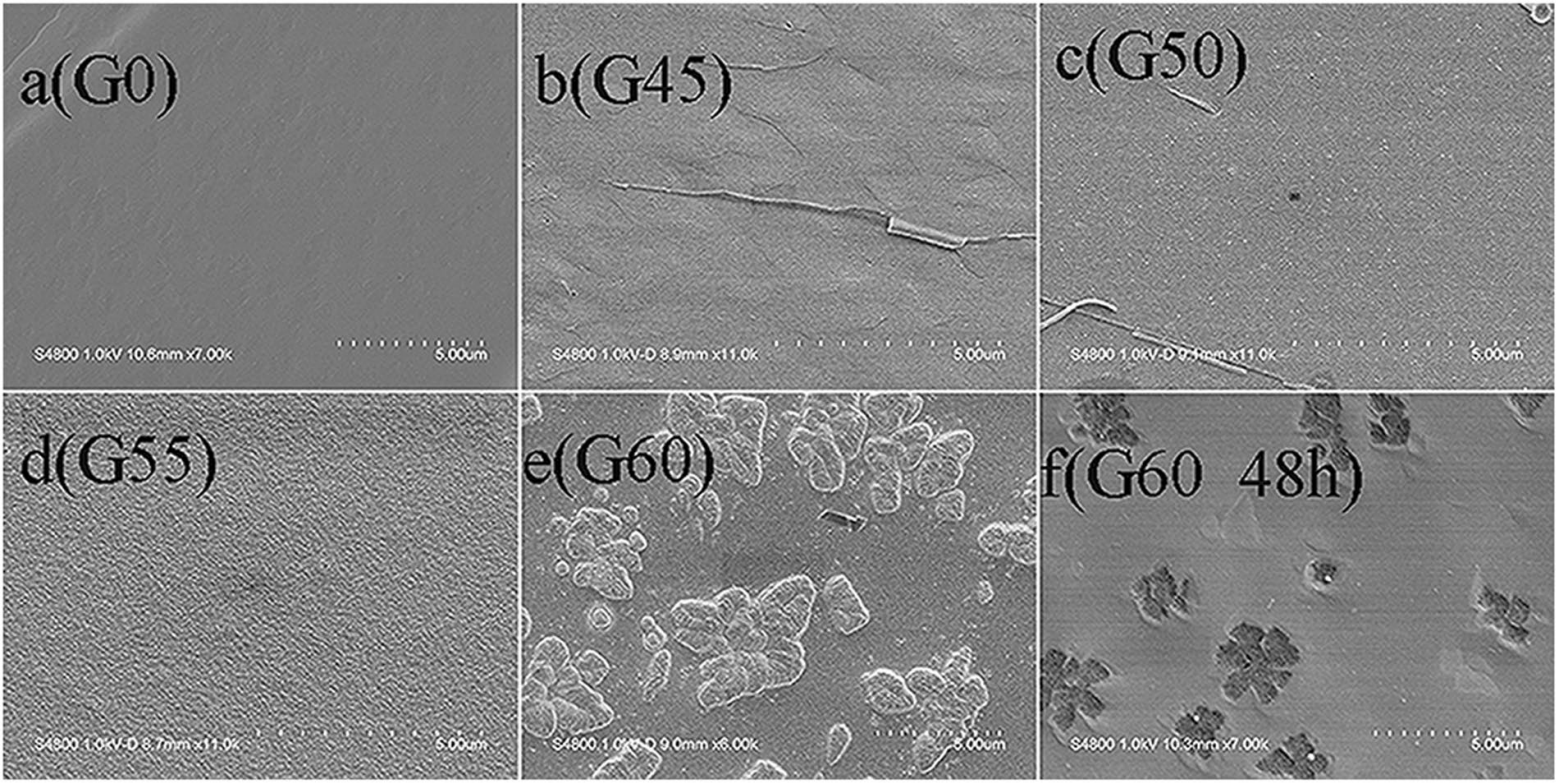
Secondary electron SEM images of all samples untreated (**a**)–(**e**) and of G60 treated in 0.1 M KNO_3_ electrolyte for 48 hrs (**f**).

**Figure 5:**
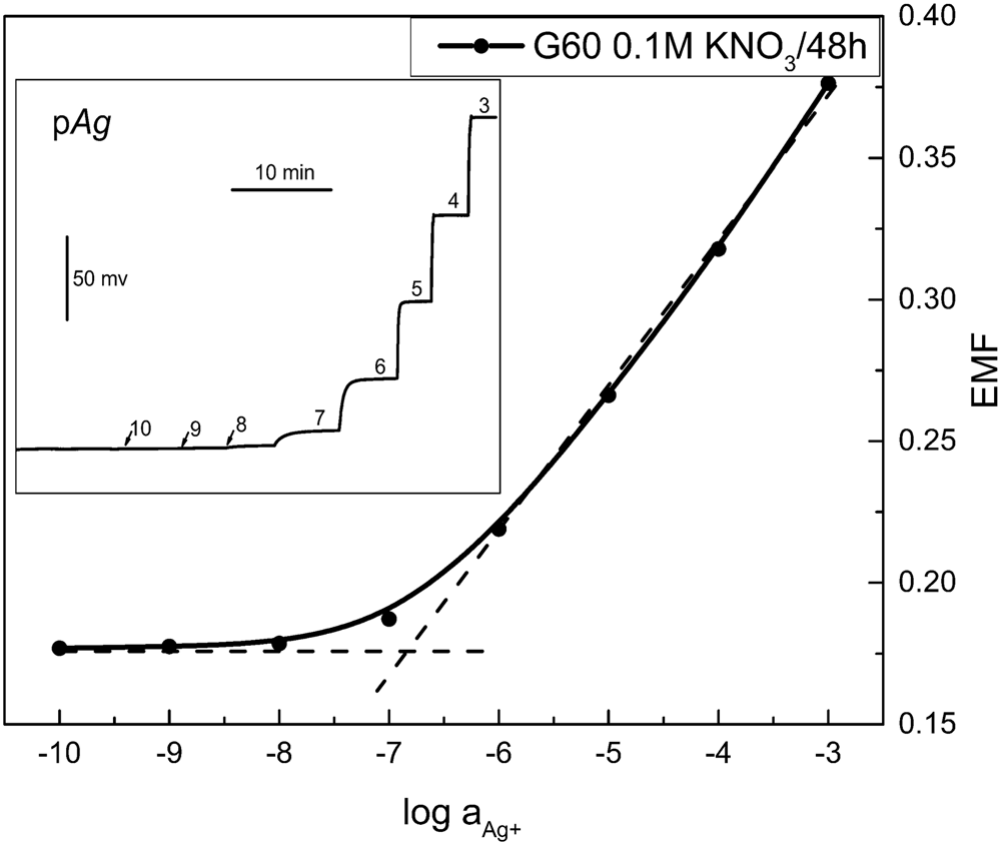
Calibration curve of G60 treated in 0.1 M KNO_3_ electrolyte solution for 48 hrs.

Another impressive observation is the super-Nernstian response behavior for the partially crystallized electrode G60. Additional SEM measurements were carried out to explore the origin of the super-Nernstian response. As shown in Fig. 4(a–d), fresh sections of G0, G45, G50 and G55 look homogeneous without indication of phase separation, while that of G60 (e) exhibits a large number of micron-sized island-like substances, which, according to XRD patterns in Fig. 1(a), belong to AgCl crystals. After soaking in a 0.1 M KNO_3_ solution for 48 hours, the island-like substances disappear (f), indicating the complete dissolution of AgCl crystals. The membrane dissolution process of G60 is also supported by the much increased double layer capacitance (CPE_dl_, Table 1) due to possible contribution of dissolution-induced capacitive reactance. For comparison with Fig. 3(e), the calibration curve of G60 after 48-hour immersion treatment was measured as shown in Fig. 5. It is obvious that after soaking the super-Nernstian response of G60 disappears, demonstrating that super-Nernstian response behavior relates to the precipitated island-like AgCl crystals. Actually, similar super-Nernstian reactivity was once reported on solid-state copper ISEs in high concentrations of chloride media^43^. It was believed that the reduction of Cu^2+^ occurred at the surface of the electrode with concomitant oxidation of the mixed sulfide electrode material when the cuprous ion was stabilized by chloride complexation, leading to super-Nernstian response. In the present case, the chloride ion concentration in the electrolyte solution was relatively high due to the dissolution of the island-like AgCl crystals on the electrode surface.Thus, at the low Ag^+^ concentration, the question arises as whether the similar chloride complexation (AgCl_n_) occurs in solution to make the EMF response to the solution activity independent on the Ag^+^ activity. Further exploration for more supporting data is needed to give an answer to this question.

### Response time and effect of pH value

Response time (RT) is defined as the time that the measured potential value reaches more than 95% of its final value (t_95%_)^44^. We measured RT of G0 and G55 electrodes which show the decreased values along with the increase of AgNO_3_ concentration and become stable in less than 5 s at the relatively high concentration (Table 3). As the ion exchange on the electrode surface is usually rapid, the RT is actually controlled by the ions transport times. Thus a longer RT at the low Ag^+^ activity could be considered due to the small diffusion driving force of the diffusion layer from which the electrode surface is separated, making the ion transport very slow^43^.

**Table 3:** Response times (t_95%_/s) of G55 and G0.

Concentration change/M	1E-10 → 1E-9	1E-9 → 1E-8	1E-8 → 1E-7	1E-7 → 1E-6	1E-6 → 1E-5	1E-5 → 1E-4	1E-4 → 1E-3
G55	256(±20)	242(±20)	145(±10)	5(±1)	5(±1)	5(±1)	5(±1)
G0	—	210(±20)	103(±10)	83(±10)	5(±1)	5(±1)	5(±1)

**Figure 6:**
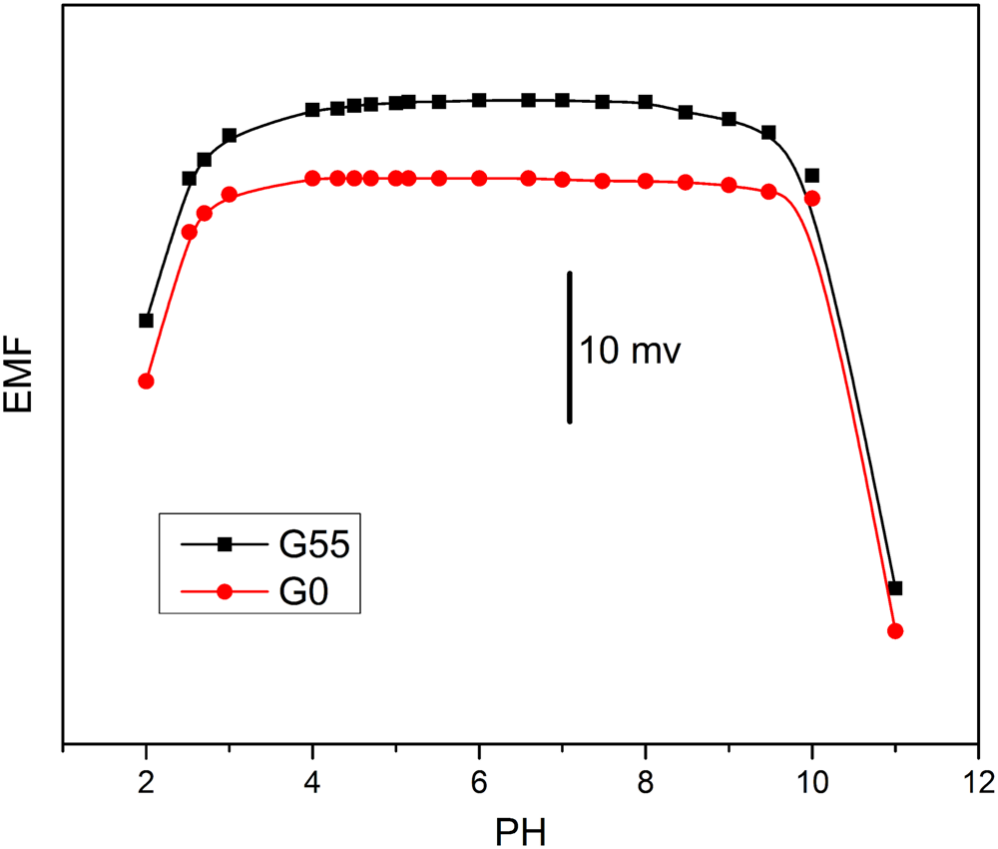
Influence of pH value on the potential of G55 and G0. Concentration of AgNO_3_: 1 mM.

The effect of pH value on electrodes G55 and G0 is also studied as shown in Fig. 6, where the pH value was adjusted with KOH and dilute HNO_3_ solutions by using a 10 μL micro-injector. The volume of the electrolyte solution can be regarded as constant due to a smaller volume of standard solution added. The final H^+^ concentration (C_H_) in the solution after each addition was calculated by the following equation:

1 $${c}_{H}={{\rm{c}}^{\prime} }_{H}+\frac{{v}_{b}\ast {c}_{b}}{V}$$

where ${{\rm{c}}^{\prime} }_{H}$ is the original concentration of H^+^ ions calculated from the original pH value, ${{\rm{v}}}_{b}$ and ${{\rm{c}}}_{b}$ are the volume and concentration of standard HNO_3_ solution added, respectively, and V is the total volume of electrolyte solution. The pH value can be calculated using the following formula:

2 $${\rm{pH}}=-\mathrm{lg}\,c({H}^{+})$$

A similar method can be used to obtain the pH value in the alkaline solution range.

It can be seen that the potential of G0 is independent of pH value in the range of 4 ≤ pH ≤ 10, while G55 shows a slightly narrower range about 4 ≤ pH ≤ 9. For G0, in fact, a lot of visible precipitants (AgOH) appear when the pH value reaches 11, corresponding to the potential reduction in the alkaline range. For G55, however, the potential is reduced when less precipitation occurs (9 ≤ pH ≤ 10), suggesting more sensitive to Ag^+^ concentration. Moreover, a monotonous reduction of electrode potentials occurs with the decreased pH value, and this is attributed to inverted-Nernstian response caused by the hydrogen ion (H^+^). A generalized model for “non-Nernstian” responses of ISEs can be used to define the response slope S as follows^45^:

3 $${\rm{S}}=\frac{RTln10}{{Z}_{I}F}(1-\frac{\partial \,\mathrm{log}\,[{I}^{{Z}_{I}}]}{\partial log{a}_{I}})$$

where a_I_ and $[{{\rm{I}}}^{{Z}_{I}}]$ are the activity of ${{\rm{I}}}^{{Z}_{I}}\,$in the sample solution and the concentration of ${{\rm{I}}}^{{Z}_{I}}\,$in the membrane phase, respectively. Z_I_ is the charge of the ${{\rm{I}}}^{{Z}_{I}}$, and the R, T, and F have their usual meanings. With the decrease in pH value, a lot of small-radius H^+^ ions enter modified surface layer^18^ (MSL) of membrane, resulting in the sharp increase in H^+^ concentration of MSL (∂log $[{{\rm{I}}}^{{Z}_{I}}]$/ ∂ log a_I_ > 1), and inducing an inverted-Nernstian response (see Eq. 3)^45^.

### Selectivity, reproducibility and stability

According to the IUPAC recommendations, the selectivity coefficients were determined with the fixed interference method (FIM)^39,46–50^. The AgNO_3_ was added to the background electrolyte solution containing constant ionic activity (1 mM) of NaNO_3_, Sr(NO_3_)_2_, Ba(NO_3_)_2_, Cu(NO_3_)_2_, Zn(NO_3_)_2_, Mn(NO_3_)_2_, Cd(NO_3_)_2_, Co(NO_3_)_2_, Hg(NO_3_)_2_ and Al(NO_3_)_3_, respectively, until a linear response slope to the Ag^+^ is observed.

The selectivity coefficients were obtained by using following equation^51^:

4 $$\mathrm{log}\,{K}_{A{g}^{+}J}^{pot}=log{a}_{A{g}^{+}}(DL)/{a}_{J}{(BG)}^{1/{Z}_{J}}$$

where ${a}_{A{g}^{+}}(DL)$ and ${a}_{J}(BG)\,\,$are activities of Ag^+^ at the detection limit and of the interfering ions in the background electrolyte solution, respectively, while Z_J_ is the charge of the interfering ion. The calculated logs of selectivity coefficients are listed in Table 4.

**Table 4:** Selectivity coefficients for silver ion-selective electrodes G55 and G0.

Selectivity coefficient		
Interfering ion	G55	G0
Na^+^	−5.05	−4.98
Sr^2+^	−5.90	−5.99
Ba^2+^	−6.32	−5.95
Cu^2+^	−5.54	−5.32
Zn^2+^	−5.96	−5.74
Mn^2+^	−6.18	−5.98
Cd^2+^	−6.20	−5.65
Co^2+^	−6.32	−6.27
Hg^2+^	−2.78	−2.73
Al^3+^	−6.94	−6.5

Results show that G55 and G0 exhibit high selectivity for Ag^+^ ion over other ions except for Hg^2+^ ion. According to previously reported work on the response of the ChG/ChH glass to Hg^2+^ ion^21,52,53^, it is believed that^21^ the Hg^2+^ ions interact with the soluble sulfide (or/and halide) formed by the membrane dissolution with subsequent formation of mercury-sulfide (or/and mercury-halide) complexes on the membrane surface, so that the electrode becomes in response to mercury ions. In other words, the detection of Ag^+^ ions by the electrode is almost impossible when a large amount of Hg^2+^ ions are co-present.

Reproducibility of the electrode performance was evaluated on two G55 samples which show a standard potential values within ±20 mV and slope values within ±2.1 mV/dec, indicating excellent reproducibility. The linear range remain unchanged after holding for 1 month, indicating good long-term stability. Moreover, the present glass ISEs are easily restored by simply polishing while retaining high reproducibility and stability.

## Conclusions

A simple fabrication procedure was used to make solid contact ion-selective electrodes based on AgCl-Ag_2_S-As_2_S_3_ ChH glass/alloy for the detection of Ag^+^ ions. The experimental results indicate that the electrode with 55 mol% AgCl has a lower detection limit than AgCl-free electrode due to increased conductivity and the more open structure. Both AgCl-free and AgCl-containing samples show a high selectivity (except for Hg^2+^), fast response time, good stability, a reasonable reproducibility, and can be used for silver ion trace detection in the appropriate conditions. Moreover, the super-Nernstian response behavior was observed from the partially crystalized sample which is related to the dissolution of island-like crystals precipitated from glass matrix.

### Data Availability

All data generated or analyzed during this study are included in this published article.
